# Burden and future projection of revision Total hip Arthroplasty in South Korea

**DOI:** 10.1186/s12891-021-04235-3

**Published:** 2021-04-22

**Authors:** Jung-Wee Park, Seok-Hyung Won, Sun-Young Moon, Young-Kyun Lee, Yong-Chan Ha, Kyung-Hoi Koo

**Affiliations:** 1 Department of Orthopaedic Surgery, Seoul National University Bundang Hospital, Seongnam, South Korea; 2 Department of Public Health Science, Complex Disease and Genome Epidemiology Branch, School of Public Health, Seoul National University, Seoul, South Korea; 3 Department of Orthopaedic Surgery, Chung-Ang University College of Medicine, Seoul, South Korea; 4 Department of Orthopaedic Surgery, Seoul National University College of Medicine, Seoul, South Korea

**Keywords:** Hip, Arthroplasty, Revision, Burden, East Asia, South Korea

## Abstract

**Background:**

The annual number of hip arthroplasties is increasing combined with the aging population worldwide. In accordance with the increasing number of primary hip arthroplasties, the number of revision total hip arthroplasties (THAs) is expected to increase. The incidence and burden of revision THAs in the United States and have been reported by registry studies. To identify potential differences according to ethnics and regional practice, it is important to obtain data from East Asia. Nevertheless, there has been a lack of studies on the burden and future projection of revision THA based on a large-scale database in East Asia**.** The purpose of this study was to evaluate annual incidence and burden of revision THAs and to project the future burden in South Korea.

**Methods:**

We identified primary THAs, primary hemiarthroplasties (HAs) and revision THAs, which were performed from 2010 to 2018, using database of Health Insurance and Review and Assessment (HIRA); nation-wide medical claim system of South Korea. The annual incidence rates (per 100,000) of primary THA, primary HA and revision THA, and the annual burden of revision THA; the number of revision THAs divided by the sum of primary hip arthroplasties and revision THAs, were calculated. The future burden of revision THAs were projected through 2030 using generalized linear model with Quasi-poisson regression.

**Results:**

During the 9-year period, the annual incidences of primary THA, primary HA and revision THA increased by 47, 29 and 3%, respectively, while the revision burden decreased from 0.13 to 0.10. Compared to 2018, the annual incidences of primary THA, HA, and revision THA were projected to increase by 7.2, 2.3 and 1.1% per year, respectively, whereas the burden of revision THA was projected to decrease to 0.07 in 2030.

**Conclusion:**

Trends of revision THA in South Korea were similar with those of national registry studies from the United States. The annual incidence of revision THA has steadily increased, whereas its burden has decreased. Findings of our study could be used for epidemiological comparison between Western countries and East Asia as well as for the establishment of medical policies of revision THA in East Asian countries.

**Supplementary Information:**

The online version contains supplementary material available at 10.1186/s12891-021-04235-3.

## Background

Total hip arthroplasty (THA) is one of the most effective orthopedic procedures and has been utilized as the treatment of choice for the patients with advanced osteoarthritis of the hip [1–4]. However, THA is associated with early and late failures and inevitably necessitates revision surgeries, which is a socioeconomic burden worldwide [5–8].

In the United States, the annual number of revision THA has steadily increased, [6, 9] but the burden of revision THA; the proportion of revision THAs to overall sum of hip arthroplasties; primary THAs, primary HAs and revision THAs, was disconcerting since 1990s [10].

There might be regional differences in the burden of revision THA according to ethnicity, implant design, fixation method; cemented versus cementless, and bearing surface [11].

Number of studies outside the United States reported the annual incidence of revision THA [12–16]. Epidemiological trend similar to the United States; increase in the number of revision THA and decrease in the burden of revision THA, was observed in other countries including Australia, New Zealand, England, Wales and Sweden except for Norway [17].

Nevertheless, there is lack of epidemiological study of revision THA in East Asia, which is necessary for regional comparison of the revision THA burden between Western countries and East Asia and for awareness of worldwide tendency.

The purpose of this study was 1) to analyze the burden of hip arthroplasty including primary THA, HA and revision THA from 2010 to 2018 and 2) to provide a future projection of these procedures to 2030 in South Korea.

## Methods

### Source of database

This is a retrospective study using secondary register-based data analysis. The Korean Health Insurance and Review and Assessment (HIRA) database includes medical claims from entire South Korean institutions. Ninety-seven percent of South Korean population is obliged to register into the Korea National Health Insurance Program (KNHIP).

The remaining 3% of the population is covered by the Medical Aid program, which is paid by the government. Data of patients under the.

Medical Aid program are submitted to HIRA in the same manner as KNHIP. Therefore, the HIRA database included the entire South Korean population.

The HIRA database contains demographics, diagnoses, procedures and prescriptions of all THA patients in South Korea.

We identified primary THAs, primary HAs and revision THAs, which had been done from January 2010 to May 2014 in South Korea, using Electronic Data Interchange (EDI) codes of THA (N0711) and hemiarthroplasty (HA) (N0715).

Since June 2014, the Korean National Health Insurance Service (NHIS) added complex surgical procedure codes (total joint arthroplasty-hip (complex) (N2070) and hemiarthroplasty-hip (complex) (N2710)) for the specific 21 complex conditions for reimbursement of an additional cost to the medical institute (Supplement. 1). THAs and HAs, which had been done from June 2014 to December 2018, were identified using 4 procedure codes (N0711, N2070, N0715, and N2710).

Revision THAs were identified using procedural codes of simple revision THA (N1711, N1721, N1715, N1725) and complex revision THA (N3710, N3720, N4710, N4720).

### Data and statistical analyses

The numbers and crude incidence rates (per 100,000) of THA, HA, and revision THA procedures were calculated according to age and gender. Annual incidence rate of each arthroplasty was calculated by dividing the number of each procedure at each year with corresponding year-specific South Korean population. The annual population data were acquired from database of the Korean Statistical Information Service. Then, the incidence rate per 100,000 person-year was calculated. The annual rates were stratified according to age and gender.

The burden of revision THA was defined as the proportion of revision THAs to overall sum of hip arthroplasties; primary THAs, primary HAs and revision THAs [18]. The future projection of THA, HA and revision THAs through 2030 were calculated by using generalized linear model with Quasi-poisson regression. Demographics-based projections through 2030 were obtained from the Korean Statistical Information Service.

## Results

### Annual incidences of primary THA, primary HA and revision THA from 2010 to 2018

From 2010 to 2018, 87,213 primary THAs, 116,079 primary HAs, and 25,232 revision THAs were done in South Korea. The number of each surgery at each year is summarized in Table 1.

**Table 1:** Change of hip arthroplasties in South Korea from 2010 to 2018

Type of Surgery	No. of procedures								
	2010	2011	2012	2013	2014	2015	2016	2017	2018
Primary THA	7657	7917	8673	9278	9520	10,357	11,036	11,227	11,548
HA	10,918	11,532	12,561	12,949	12,941	13,737	13,420	13,613	14,408
Revision THA	2728	2671	2777	2701	2752	2826	2875	3032	2870
Population	50,515,666	50,734,284	50,948,272	51,141,463	51,327,916	51,529,338	51,696,216	51,778,544	51,826,059
Primary THA incidence	15.16	15.60	17.02	18.14	18.55	20.10	21.35	21.68	22.28
HA incidence	21.61	22.73	24.65	25.32	25.21	26.66	25.96	26.29	27.80
Revision THA incidence	5.40	5.26	5.45	5.28	5.36	5.48	5.56	5.86	5.54
Burden of revision THA	0.13	0.12	0.12	0.11	0.11	0.10	0.10	0.10	0.10

**Fig. 1:**
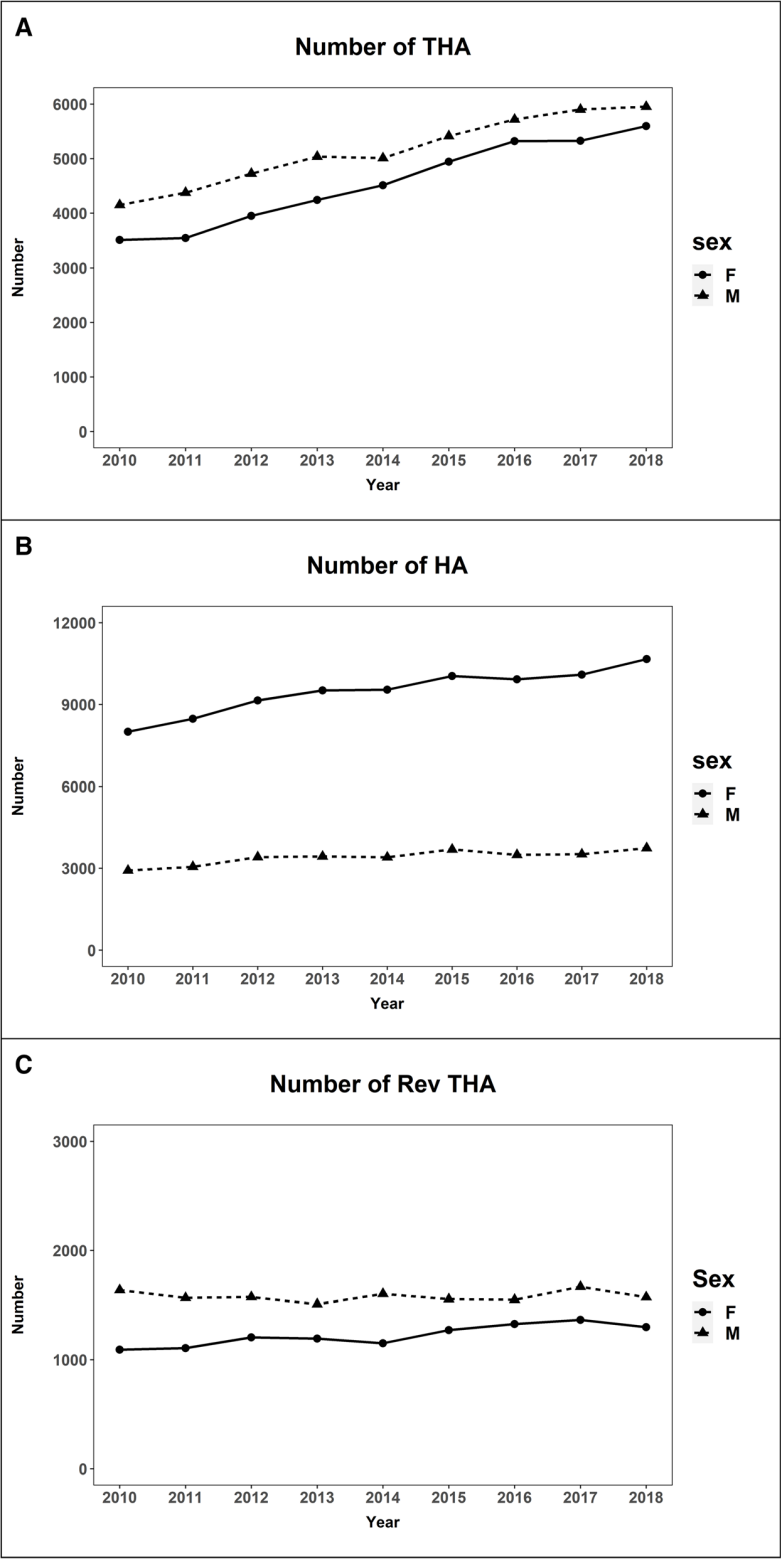
Number of primary total hip arthroplasty **a**, hemiarthroplasty **b**, and revision total hip arthroplasty **c** in each gender (THA, total hip arthroplasty; HA, hemiarthroplasty; RevTHA, revision total hip arthroplasty)

During the 9-year period, the annual crude incidence of primary THA and that of primary HA increased by 47% (15.2/100,000 in 2010 to 22.3/100,000 in 2018) and 29% (21.6/100,000 in 2010 to 27.8/100,000 in 2018), respectively. Nevertheless, the annual incidence rate of revision THA remained stable at an average of 5.5/100,000 (range, 5.4–5.9/100,000) (Fig. 1), and the burden of revision THA decreased from 0.13 to 0.10 (Table 1).

Overall, 86% of primary HA patients were ≥ 70 years of age, whereas 50% of primary THA patients and 29% of revision THA patients were < 60 years of age (Table 2).

**Table 2:** Demographic characteristic of patients who had hip replacements from 2010 to 2018 in Korea

	Primary THA (*n* = 87,213)	HA (*n* = 116,079)	Revision THA (*n* = 25,232)
Gender (%)			
Male	46,276 (53%)	30,664 (26%)	14,224 (56%)
Female	40,937 (47%)	85,415 (74%)	11,008 (44%)
Age category			
10–19 years	156	24	21
20–29 years	1952	71	102
30–39 years	6628	330	540
40–49 years	12,639	1069	1995
50–59 years	21,968	3512	4600
60–69 years	20,807	11,270	6975
70–79 years	18,000	42,723	7757
≥ 80 years	5063	57,080	3109

*THA* total hip arthroplasty, *HA* hemiarthroplasty

**Fig. 2:**
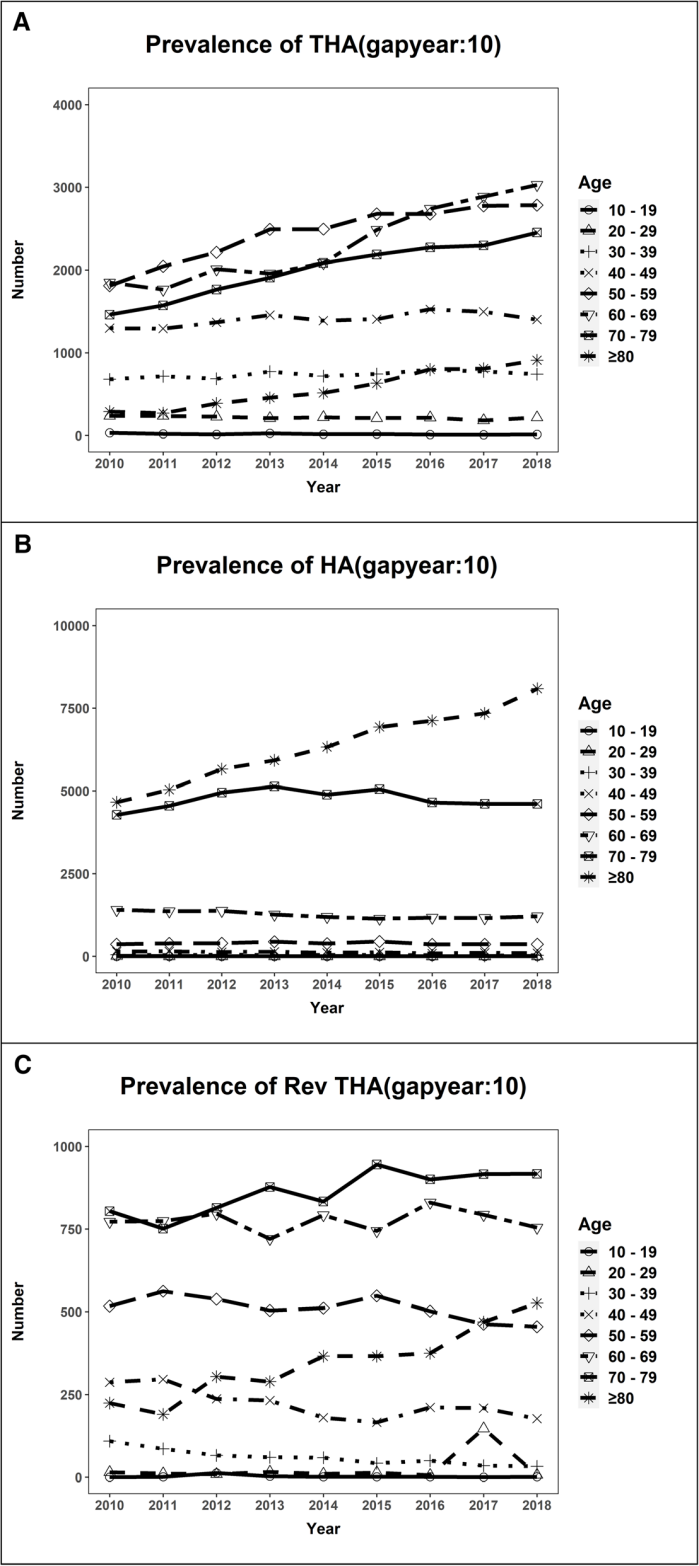
Number of total hip arthroplasty **a**, hemiarthroplasty **b**, and revision total hip arthroplasty **c** stratified by age group from 2010 to 2018 (THA, total hip arthroplasty; HA, hemiarthroplasty; RevTHA, revision total hip arthroplasty)

The annual number of primary THA increased in age groups of 50–59 years, 60–69 years, 70–79 years and ≧ 80 years, whereas the annual number of HA increased only in the age group of ≧80 years. The annual number of revision THA slightly increased in age groups of 70–79 years and ≧ 80 years (Fig. 2).

**Fig. 3:**
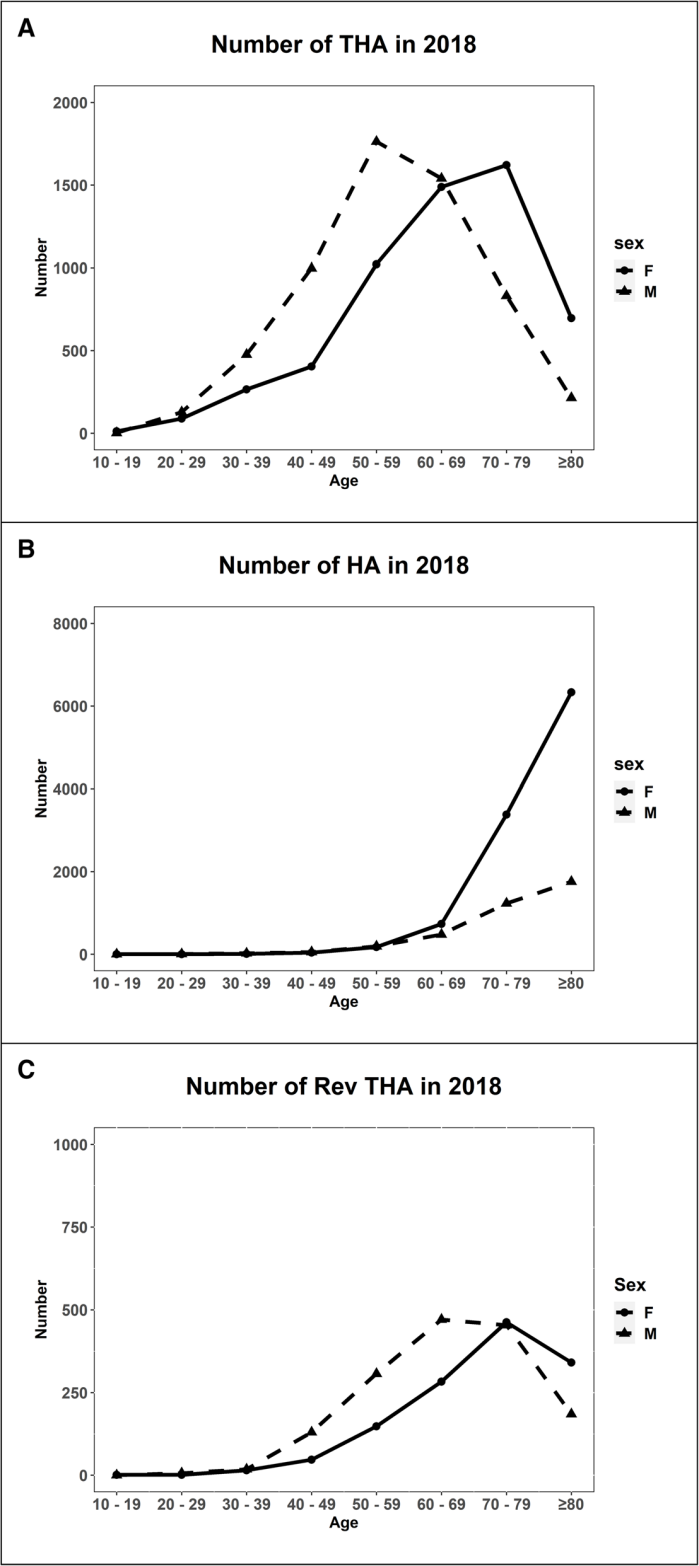
Number of total hip arthroplasty **a**, hemiarthroplasty **b**, and revision total hip arthroplasty **c** in each age group in 2018 (THA, total hip arthroplasty; HA, hemiarthroplasty; RevTHA, revision total hip arthroplasty)

In 2018, the peak age of primary THA was 50–59 years in men and 70–79 years in women. The number of HA abruptly increased after 50 years, especially in women. The peak age of revision THA was 60–69 years in men and 70–79 years in women (Fig. 3).

The burden of revision THA slightly decreased from 0.13 in 2010 to 0.10 in 2018.

### Projection of annual numbers of primary THA, primary HA and revision THA to 2030

**Fig. 4:**
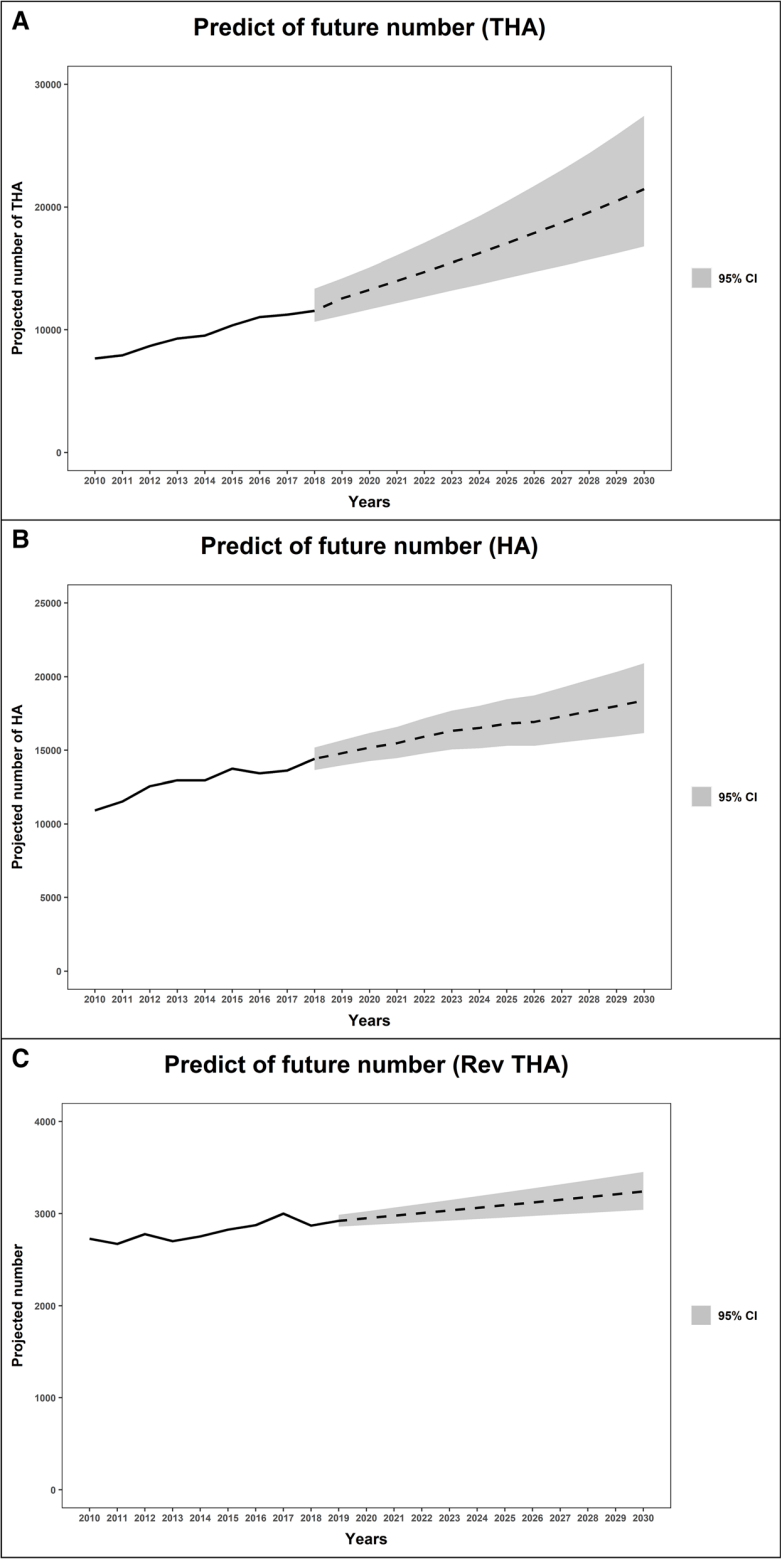
Projection of future cases of total hip arthroplasty **a**, hemiarthroplasty **b**, and revision total hip arthroplasty **c** in South Korea (THA, total hip arthroplasty; HA, hemiarthroplasty; RevTHA, revision total hip arthroplasty)

In our prediction model, the numbers of primary THA and HA were predicted to increase to 21,465 (95% confidence interval (CI) 16,792 – 27,451) and 18,384 (95% CI 16,162 – 20,918), respectively, while the number of revision THA was predicted to be 3241 (95% CI 3042 – 3454) in 2030 (Fig. 4). Compared to 2018, the annual incidences of primary THA, HA, and revision THA were projected to increase by 7.2, 2.3 and 1.1% per year, respectively, whereas the burden of revision THA was projected to decrease to 0.07 in 2030.

## Discussion

Our study showed that the annual crude incidences of primary THA and primary HA increased by 47 and 29%, respectively, whereas and revision THA increased by only 3% and the burden of revision THA decreased from 0.13 to 0.10 during the period from 2010 to 2018 in South Korea. The burden of revision THA was projected to decrease to 0.07 in 2030.

These trends were similar with those of previous national registry studies from the United States, United Kingdom and Taiwan (Table 3) [9, 13, 14, 19, 20].

**Table 3:** Change of burden of revision total hip arthroplasty in each country

Author	Country	Study period	Number of THA	Number of revision THA	Ratio of revision THA
Wolf BR [19]	USA	1991–2008	188,646 to 279,627	48,528 to 57,331	0.20 to 0.17
Kumar A [8]	Taiwan	1998–2009	3726 to 4972	931 to 968	0.20 to 0.16
Kurtz SM [10]	USA	2005–2015	231,648 to 378,089	42,451 to 55,647	0.15 to 0.13
Bozic KJ [2]	USA	2006–2010	222,239 to 291,994	40,555 to 49,857	0.15 to 0.15
Patel A [15]	England & Wales	2008–2012	71,077 to 79,949	7038 to 10,008	0.09 to 0.11
Current study	Korea	2010–2017	7657 to 11,227	2728 to 3032	0.13 to 0.10

In the study of Wolf et al. the revision burden reduced from 0.20 in 1991–1993 to 0.17 in 2006–2008 [19]. Another study by Schwartz et al. showed similar decrease of the revision burden from 0.19 in 2001 to 0.13 in 2010 [21].

In 2014, Kurtz et al. estimated hip arthroplasty data of from the Nationwide Inpatient Sample in United States and projected the number of hip arthroplasties and the burden of revision THA. In their study, the number of primary THA and that of revision THA increased by 6.0% and by 10.8%, respectively, and the revision burden increased from 0.135 to 0.141 from 2009 to 2010. The numbers were expected to increase by 74% for primary THA and 36% for revision THA, but the revision burden was projected to decrease from 0.14 to 0.11 between 2010 and 2020 [9].

In 2019, Heckmann et al. compared the hip arthroplasty data from American Joint Replacement Registry (AJRR) to national registry data of Australia, New Zealand, England, Wales, Sweden and Norway [17]. In the AJRR, the burden of revision THA decreased from 0.14 in 2014 to 0.09 in 2016. The burden of revision THA decreased in most counties except for Norway. The burden decreased from 0.12 to 0.11 in Australia, from 0.15 to 0.12 in New Zealand, from 0.10 to 0.08 in England/Wales, and from 0.11 to 0.10 in Sweden [17]. However, in Norway, the burden slightly increased from 0.135 to 0.138 [17].

As opposed to the study of Heckmann et al., Patel et al. reported an increase of revision THA burden in England and Wales from 0.09 in 2008 to 0.11 in 2012 [14].

The annual incidence rate (per 100,000 person-year) of primary THA and revision THA increased from 101 to 134 and from 19.2 to 21.1 per 100,000 Danish inhabitants from 1996 to 2002, respectively. The incidence rates of primary THA and revision THA increased by 30 and 10% during the same period [15]. In line with this finding, the incidence of THA has also increased in South Korea during last decade. Aging of Korean population could be one of the main reasons of our findings.

In a previous study of THA epidemiology from South Korea, both the number and incidence rate of primary hip arthroplasty increased steadily from 2007 to 2011, but there was no significant change in the number of revision THA during the period [16].

The worldwide trend of decrease in the burden of revision THA (Table 3) means relatively small increase in the number of revision THAs compared to a substantial growth in the number of primary hip arthroplasties.

There might be some explanations for this decrease of the revision burden. First, surgical technique for primary THA has been improved with time [22]. Second, modern technologies of hip prostheses, especially, bearing surfaces might have prolonged the longevity of primary THA [23].

The strength of our study was that the study is based on national registry data. The results cover nearly 100% of all hip arthroplasties in South Korea. Such data are only available in few countries. Our study was based on national registry data and has inherent limitation. We could not identify the reason of arthroplasty, type of original prosthesis, type of fixation; cementless versus cemented, bearings surfaces and the reason of revision surgery in patients undergoing revision THA, because of de- identification of the HIRA database.

## Conclusions

In this study, we confirmed an increase in number and incidence of revision THA as well as primary THA and HA from 2010 to 2018 in South Korea. The burden of revision THA in that period gradually decreased from 0.13 to 0.10, which was similar to reports from Western countries.

## Supplementary Information

**Additional file 1.** Complex conditions for reimbursement in arthroplasty in South Korea.

## Data Availability

The datasets used and/or analyzed during the current study are available from the Korean Health Insurance and Review and Assessment (HIRA) database. https://opendata.hira.or.kr/home.do

## Abbreviations

THA: Total hip arthroplasty
HA: Hemiarthroplasty
HIRA: Health Insurance and Review and Assessment
KNHIP: Korea National Health Insurance Program
EDI: Electronic Data Interchange
NHIS: National Health Insurance Service
CI: Confidence interval
AJRR: American Joint Replacement Registry

## References

[1] Berry DJ, Harmsen WS, Cabanela ME, Morrey BF (2002). Twenty-five-year survivorship of two thousand consecutive primary Charnley total hip replacements: factors affecting survivorship of acetabular and femoral components. J Bone Joint Surg Am.

[2] Learmonth ID, Young C, Rorabeck C (2007). The operation of the century: total hip replacement. Lancet.

[3] Soderman P, Malchau H, Herberts P (2000). Outcome after total hip arthroplasty: part I. general health evaluation in relation to definition of failure in the Swedish National Total hip Arthoplasty register. Acta Orthop Scand.

[4] Soderman P, Malchau H, Herberts P, Zugner R, Regner H, Garellick G (2001). Outcome after total hip arthroplasty: part II. Disease-specific follow-up and the Swedish National Total hip Arthroplasty Register. Acta Orthop Scand.

[5] Kurtz SM, Ong KL, Schmier J, Mowat F, Saleh K, Dybvik E, Karrholm J, Garellick G, Havelin LI, Furnes O, Malchau H, Lau E (2007). Future clinical and economic impact of revision total hip and knee arthroplasty. J Bone Joint Surg Am.

[6] Ong KL, Mowat FS, Chan N, Lau E, Halpern MT, Kurtz SM (2006). Economic burden of revision hip and knee arthroplasty in Medicare enrollees. Clin Orthop Relat Res.

[7] Rudasill SE, Ng A, Kamath AF (2018). Preoperative serum albumin levels predict treatment cost in Total hip and knee Arthroplasty. Clin Orthop Surg.

[8] Im C, Lee KJ, Min BW, Bae KC, Lee SW, Sohn HJ (2018). Revision Total hip Arthroplasty after ceramic bearing fractures in patients under 60-years old; mid-term results. Hip Pelvis.

[9] Kurtz SM, Ong KL, Lau E, Bozic KJ (2014). Impact of the economic downturn on total joint replacement demand in the United States: updated projections to 2021. J Bone Joint Surg Am.

[10] Kurtz S, Mowat F, Ong K, Chan N, Lau E, Halpern M (2005). Prevalence of primary and revision total hip and knee arthroplasty in the United States from 1990 through 2002. J Bone Joint Surg Am.

[11] Hoaglund FT, Oishi CS, Gialamas GG (1995). Extreme variations in racial rates of total hip arthroplasty for primary coxarthrosis: a population-based study in San Francisco. Ann Rheum Dis.

[12] Ingvarsson T, Hagglund G, Jonsson H, Lohmander LS (1999). Incidence of total hip replacement for primary osteoarthrosis in Iceland 1982-1996. Acta Orthop Scand.

[13] Kumar A, Tsai WC, Tan TS, Kung PT, Chiu LT, Ku MC (2015). Temporal trends in primary and revision total knee and hip replacement in Taiwan. J Chin Med Assoc.

[14] Patel A, Pavlou G, Mujica-Mota RE, Toms AD (2015). The epidemiology of revision total knee and hip arthroplasty in England and Wales: a comparative analysis with projections for the United States. A study using the National Joint Registry dataset. Bone Joint J.

[15] Pedersen AB, Johnsen SP, Overgaard S, Soballe K, Sorensen HT, Lucht U (2005). Total hip arthroplasty in Denmark: incidence of primary operations and revisions during 1996-2002 and estimated future demands. Acta Orthop.

[16] Yoon PW, Lee YK, Ahn J, Jang EJ, Kim Y, Kwak HS, Yoon KS, Kim HJ, Yoo JJ (2014). Epidemiology of hip replacements in Korea from 2007 to 2011. J Korean Med Sci.

[17] Heckmann N, Ihn H, Stefl M, Etkin CD, Springer BD, Berry DJ, Lieberman JR (2019). Early results from the American joint replacement registry: a comparison with other National Registries. J Arthroplasty.

[18] Malchau H, Herberts P, Eisler T, Garellick G, Soderman P (2002). The Swedish Total hip replacement register. J Bone Joint Surg Am.

[19] Wolf BR, Lu X, Li Y, Callaghan JJ, Cram P (2012). Adverse outcomes in hip arthroplasty: long-term trends. J Bone Joint Surg Am.

[20] Bozic KJ, Kamath AF, Ong K, Lau E, Kurtz S, Chan V, Vail TP, Rubash H, Berry DJ (2015). Comparative epidemiology of revision Arthroplasty: failed THA poses greater clinical and economic burdens than failed TKA. Clin Orthop Relat Res.

[21] Schwartz BE, Piponov HI, Helder CW, Mayers WF, Gonzalez MH (2016). Revision total hip arthroplasty in the United States: national trends and in-hospital outcomes. Int Orthop.

[22] Knight SR, Aujla R, Biswas SP (2011). Total hip Arthroplasty - over 100 years of operative history. Orthop Rev (Pavia).

[23] Hamilton WG, McAuley JP, Dennis DA, Murphy JA, Blumenfeld TJ, Politi J (2010). THA with Delta ceramic on ceramic: results of a multicenter investigational device exemption trial. Clin Orthop Relat Res.

