# Supplementary material for: Burden and future projection of revision Total hip Arthroplasty in South Korea
Source: BMC Musculoskelet Disord. 2021 Apr 22;22:375. doi: 10.1186/s12891-021-04235-3 (PMC8063384; doi:10.1186/s12891-021-04235-3)
Supplement: Supplementary file 1 — Additional file 1. Complex conditions for reimbursement in arthroplasty in South Korea. [file 12891_2021_4235_MOESM1_ESM.docx]

**Supplement**

**Supplement 1. Complex conditions for reimbursement in arthroplasty in South Korea.**

| **Criteria for complex conditions** |
| --- |
| (1) Objective evidence of following diseases by consultation to specialists in each field (subspecialtists in internal medicine) |
| - 1. Patients with chronic renal failure |
| - 1. Patients who have received or need organ transplantation |
| - 1. Patients taking anticoagulants because of the cardiovascular stents |
| - 1. Patients with severe myocardiac infarction / angina – Goldman cardiac risk III or more |
| - 1. Patients with uncontrolled diabetes mellitus (HbA1C > 7.0) |
| - 1. Patients with liver cirrhosis |
| - 1. Patients with hematologic malignancy |
| - 1. Patients with hemophilia or coagulopathy |
| - 1. Patients with severe obstructive lung disease (FEV1 under 50%) |
| - 1. Patients who have past medical history of treating venous thromboembolism |
| - 1. Patients taking anticoagulants more potent than aspirin due to stroke or other diseases |
| 1. Patients under treatment for rheumatic disease (DAS 28 > 5.1) |
| 1. Patients with peripheral arterial obstructive disease |
| 1. Patients with progressive spinal cord palsy or cauda equine syndrome |
| 1. Patients with pathologic fractures : primary bone cancer, metastatic bone cancer or osteoporosis |
| 1. Arthroplasty following sequelae of septic arthritis or periprosthetic joint infection |
| 1. Arthroplasty on more than 1 inch of bone loss in longitudinal axis |
| 1. Arthroplasty on more than 15° of bone deformity |
| 1. Revision arthroplasty following pseudoparalysis, cuff tear arthroplasty, wide tear of rotator cuff |
| (10) Joint contracture over 20° |
| (11) Re-revision arthroplasty after revision arthroplasty |
